# The effects of laser acupuncture dosage at PC6 (Neiguan) on brain reactivity: a pilot resting-state fMRI study

**DOI:** 10.3389/fnins.2023.1264217

**Published:** 2023-10-12

**Authors:** Yi-Chuan Chang, Chun-Ming Chen, Ing-Shiow Lay, Yu-Chen Lee, Cheng-Hao Tu

**Affiliations:** ^1^Graduate Institute of Acupuncture Science, College of Chinese Medicine, China Medical University, Taichung, Taiwan; ^2^Department of Chinese Medicine, China Medical University Beigang Hospital, Yunlin, Taiwan; ^3^Department of Medical Imaging, China Medical University Hospital, Taichung, Taiwan; ^4^School of Post-Baccalaureate Chinese Medicine, China Medical University, Taichung, Taiwan; ^5^School of Chinese Medicine, China Medical University, Taichung, Taiwan; ^6^Department of Chinese Medicine, China Medical University Hospital, Taichung, Taiwan

**Keywords:** laser acupuncture, Neiguan, rostral ventrolateral medulla, resting-state functional magnetic resonance imaging, functional connectivity

## Abstract

Previous studies indicated that laser acupuncture (LA) may effectively treat various medical conditions. However, brain responses associated with LA intervention have not been fully investigated. This study is focused on the effect of LA with different energy density (ED) in brain using resting-state functional magnetic resonance imaging (fMRI). We hypothesized that different ED would elicit various brain responses. We enrolled healthy adults participants and selected bilateral PC6 (Neiguan) as the intervention points. LA was applied, respectively, with ED of 0, 7.96, or 23.87 J/cm^2^. Two 500-s resting-state fMRI scans were acquired before and after intervention, respectively. The functional connectivity (FC) was calculated between autonomic nerve system-regulation associated brainstem structures and other brain regions. Compared to other dosages, the FC between rostral ventrolateral medulla and orbitofrontal cortex has more enhanced; the FC between caudal ventrolateral medulla, nucleus of the solitary tract/nucleus ambiguus, and dorsal motor nucleus of the vagus and somatosensory area has more weakened when ED was 23.87 J/cm^2^. Different dosages of LA have demonstrated varied regions of FC changes between regions of interest and other brain areas, which indicated that variations in EDs might influence the clinical efficacy and subsequent impacts through distinct neural pathways within the brain.

## 1. Introduction

Laser acupuncture (LA) is a form of low-level laser therapy (LLLT) that has gained popularity in clinical practice due to its non-invasive, non-thermal, painless, and bloodless nature (Wu et al., 2009; Chen et al., 2019). Contrary to conventional acupuncture, LA employs low-power laser light, typically ranging from 1 mW to 500 mW. This approach combines the attributes of LLLT to facilitating tissue regeneration, diminishing inflammation, and mitigating pain (Huang et al., 2009). Additionally, LA incorporates acupuncture treatment principles to target specifically regions or acupoints based on distinct indications. LA offers several advantages such as flexibility in adjusting the exposed zone, accurate dosing of exposure, and the ability to combine the technique with any other type of treatment (Moskvin and Agasarov, 2020). Despite the increasing use of LA and a notable therapeutic efficacy, there is currently no general consensus on the intervention strategy for operators in clinical treatment.

Nevertheless, an increasing body of literature has demonstrated that various energy densities (ED) of LA stimulation follow Arndt–Schulz law and can produce distinct effects or with biphasic response (Huang et al., 2009). Some published paper suggested to control ED between 4 and 10 J/cm^2^ for the target tissue (Zein et al., 2018). Additionally, a review article has highlighted that different radiant exposures of ED dosage may have varying biological effects, and the ED around 10 J/cm^2^ can also result in diverse or opposite biological reactivity (Cronshaw et al., 2019). For example, some studies have pointed out that LA tends to increase sympathetic and inhibit parasympathetic nerve activities in patients with insomnia (Chen et al., 2019), but others have found the opposite performance in LA interventions for night shift worker groups by different LA intervention and parameter settings (Wu et al., 2009). Previous research conducted by our team has demonstrated that LA can effectively stimulate the Neiguan acupoint (PC6) with varying EDs, resulting in a biphasic dose–response effect on the autonomic nervous system (ANS) (Chang et al., 2022).

Despite the increasing awareness and publication of the varying biological effects induced by LA stimulation with different EDs, there has been limited discussion regarding the effects of differential stimulations on corresponding regions of the brain, such as the regions in medulla that responsible for regulating the ANS, or the functional connectivity (FC) between these region and various parts of the brain after LA intervention. The stimulation of PC6 has already been demonstrated in a published article to modulate the amplitude of the intrinsic cortical activity in the brain (Zhang et al., 2012). Several functional magnetic resonance imaging (fMRI) studies have shown that acupuncture at PC6 can change neural activity not only in pain-related areas such as the thalamus (Napadow et al., 2008), hypothalamus (Bai et al., 2010), midbrain periaqueductal gray (PAG) (Dhond et al., 2008), and in the anterior cingulate cortex (ACC) (Hohenschurz-Schmidt et al., 2020), but also structures closely associated with heart rate variability (HRV), such as the rostroventral medulla (RVM) (Napadow et al., 2005). Nonetheless, the majority of findings pertaining to brain activity responses are rooted in conventional acupuncture or electroacupuncture investigations. Research focused on LA, particularly concerning various EDs, remains conspicuously limited within the realm of fMRI-related studies.

Furthermore, changes in ANS and sympathovagal balance are mainly related to the rostral ventrolateral medulla (RVLM) in medulla. The RVLM is considered as one of the main structures responsible for regulating the sympathetic nervous system (SNS) in response to maintaining a constant heartbeat and blood pressure regulation of the ejection mechanism. Besides, the RVLM also receives signals from the caudal ventrolateral medulla (CVLM) to balance parasympathetic activity. The CVLM activity is captured by the nucleus of the solitary tract (NTS), and NTS affects the parasympathetic nervous system (PNS) through the dorsal motor nucleus of the vagus (DMV) and nucleus ambiguus (NA) (Albaghdadi, 2007). Considering that LA stimulation with different EDs has been found to produce different biological effects, this study aims to investigate the relationship between stimulation with various EDs and how changes in various ED stimulation affect the FC between specific regions of interest (ROIs) in the medulla and various other parts of the brain. To achieve this objective, we choose PC6 as the stimulation intervention point with different EDs, and utilized resting-state fMRI scan to explore the potential changes of FC in different brain regions. We hypothesis that the FC changes in response to LA stimulation with different EDs may vary in different brain regions, and the effects of LA may affect individuals through different nerve pathways in central nervous system (CNS) after stimulation with different EDs.

## 2. Materials and methods

### 2.1. Ethics approval

The implementation of this clinical trial program abides by the Declaration of Helsinki, stipulated by the World Medical Association, so as to ensure the life and safety of clinical trial subjects’ health, personal privacy, and dignity. Furthermore, the research project had approved by the Research Ethics Committee of China Medical University and hospital, with the case number CMUH110-REC2-252, prior to commencement. The protocol had also been registered at ClinicalTrials.gov (Identifier: NCT05458713).

### 2.2. Participants

In this pilot study, our primary focus is to investigate the potential cerebral responses of individuals with normal health to diverse LA EDs. To mitigate the potential influence of cardiovascular disease risk factors on study, we recruited healthy male and female, with postmenopausal female participants excluded because of the markedly heightened risk linked to cardiovascular disease (El Khoudary et al., 2020), subjects between the ages of 18 to 55 years with a body mass index (BMI) ranging from 18.5 to 24 kg/m^2^. To ensure the safety of participants and the stability of data collection, other exclusion criteria included: (1) subjects with chronic diseases such as coronary artery disease, hypertension, diabetes mellitus, hyperlipidemia, and history of cancer; (2) subjects experiencing recent carpal tunnel syndrome or similar symptoms; (3) subjects with functional or organic diseases related to the CNS; (4) subjects unable to follow instructions or comply with study requirements; (5) pregnant women or subjects with metal implants or pacemakers; (6) subjects who had taken nerve activity-inhibiting medications such as psychiatric medications or analgesics within the past 3 months; and (7) subjects with open wounds or scar tissue around the PC6 area that may interfere with the LA intervention.

### 2.3. Study design

**Figure 1:**
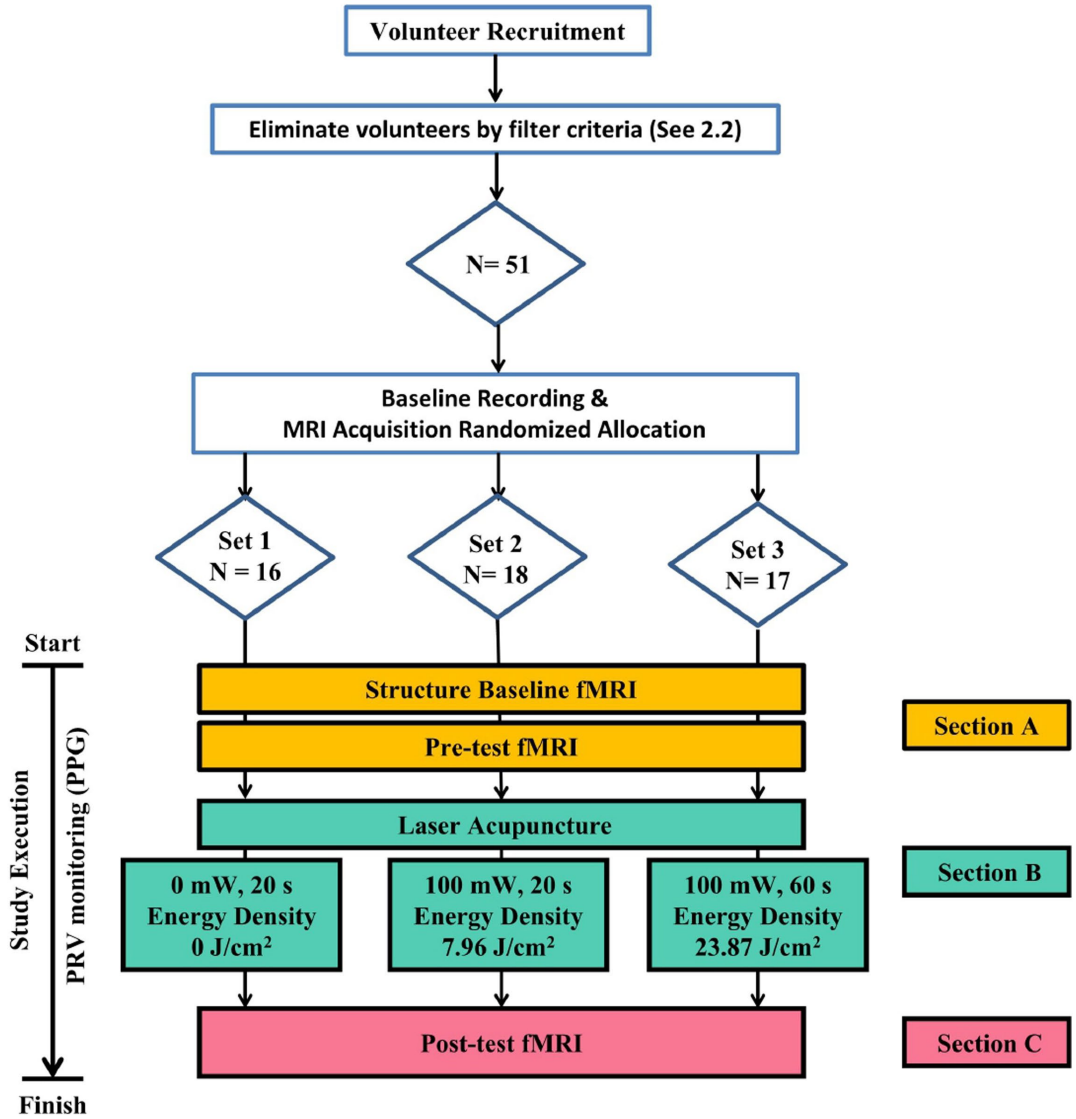
Flowchart of Experiment. Eligible subjects meeting screening criteria are randomly grouped via a random number table to receive laser acupuncture with varying energy densities in a three-section trial.

In this study, the experiment has been designed with 1 control group and 2 experimental groups, each group comprising varying ED of LA. Upon random assignment of participants to respective groups using a random number table generated by Excel (Microsoft Inc., United States), the participants were subjected to execute bilateral PC 6 LA stimulation and undergo resting-state fMRI scanning before and after the intervention. The MRI built-in photoplethysmography (PPG) equipment was utilized to monitor the subject’s vital signs such as pulse rate. The purpose of these settings was to facilitate the acquisition of spontaneous brain activity during the resting state, and the obtained data was analyzed to assess the alterations in brain activity pre- and post-intervention. The experimental protocol is depicted in Figure 1.

The experimental protocol comprises 3 distinct sections, during which the participants undergo a total of three sessions of fMRI scans. In Section A, the patient is positioned supine on the fMRI platform with the head secured in place. This section involves the execution of a structural scan and a functional scan. The purpose of the whole-brain anatomical image scan is to verify the absence of any structural abnormalities within the brain existed. Section B involves the administration of the LA intervention, whereas Section C comprises the third fMRI scan. This scan is performed after the LA intervention to enable the comparison of effects before and after the intervention.

### 2.4. Laser acupuncture intervention

The LA equipment used in this project was the RJ laser (Handylaser Trion; Reimers and Janssen GmbH, Winden, Germany). The complete details of the LA parameter set-tings are presented in Table 1. The 3 groups were subjected to the same oscillation/resonance frequency, irradiation area, and other relevant parameters. With the exception of control group Set 1, which received no actual energy input, experimental groups Set 2 and Set 3 attained EDs of 7.96 J/cm^2^ and 23.87 J/cm^2^ respectively, by varying the duration of exposure time in 20 and 60 s.

**Table 1:** Parameters of the laser device with different intervention Sets of the groups.

Laser device		Gallium-aluminum-arsenide (GaAIAs) infrared laser RJ laser				
Wavelength		810 nm				
Intervention mode		Contact				
Frequency		Pulsed wave at Bahr 2: 1199 Hz for central/middle tissue layer				
Intervention region		Bilateral PC6				
Set	Probe Size	Output Power	Exposure Time	Single Joule	Total Joule	Energy density
1	4 mm	0 mW	20 s	0 J	0 J	-------------
2	4 mm	100 mW	20 s	1 J	2 J	7.96 J/cm^2^
3	4 mm	100 mW	60 s	3 J	6 J	23.87 J/cm^2^

LA was administered bilaterally at the PC6 acupoints (two inches above the transverse crease of the wrist, about 5 cm or the width of three fingers, between the palmaris longus muscle and the flexor carpi radialis tendon) (World Health Organization. Regional Office for the Western Pacific, 2008). Furthermore, the LA procedures was performed by a qualified LA expert practitioner who has received extensive training in traditional Chinese medicine (TCM) and traditional acupuncture with 8 years and has over two years of clinical experience in LA.

### 2.5. Image acquisition

FMRI imaging was conducted utilizing a 3 T magnetic resonance imaging system (SIGNA Architect AIR Edition, GE healthcare, Chicago, IL, United States) housed in the Medical Imaging Department of the China Medical University-affiliated hospital. To enter the scanning room, participants must discard all metallic and magnetic objects (such as credit cards, magnetic buckles, watches, jewelry, etc.) and utilize earplugs to safeguard their ears. During the scan, participants are instructed to remain still with their eyes opened and to relax without any movement. Following positioning, the magnetic field gradient is shimming automatically, and tri-pilot images are acquired to determine the field of view’s position. In Section A, a whole-brain anatomical image scan is performed approximately in 5 min with an 3-dimensional spoiled gradient echo sequence (repetition time: 7.356 ms; echo time: 2.736 ms; flip angle = 12°; matrix = 224 × 224 × 170; field of view = 224 × 224 × 170 mm^3^), followed by a resting-state functional MRI scan before LA stimulation with 200 continuous whole-brain scans using an ascending interleaved echo-planar imaging (EPI) sequence, each lasting 2.5 s, total duration: 8 min and 40 s (repetition time = 2,500 ms; echo time = 30 ms; flip angle = 90°; matrix = 64 × 64; field of view = 224 × 224 mm^2^; slice number = 40; and slice thickness = 3.5 mm). After receiving LA stimulation in Section B, the participants undergo another resting-state fMRI scan in Section C. Experimental process would suspend in the event of subject discomfort occurring during any section.

### 2.6. Image data pre-processing

For image data preprocessing, Data Processing Assistant for Resting-State fMRI (DPARSF, State Key Laboratory of Cognitive Neuroscience and Learning, Beijing Normal University, China) was utilized, which been written on the Matlab 2021b (Mathworks Inc., Sherborn, MA, United States) library. The fMRI images were first corrected for slice acquisition times and realignment with other images to minimize the effects of head movement during scanning. Afterward, the EPI template in DPARSF was utilized for the spatial normalization of functional images. The normalized fMRI images were smoothed with a three-dimensional Gaussian kernel (8-mm full width at half maximum). Finally, in the preprocessed images, voxel-wise brain activities were detrended linearly, confounding variables such as six head movement parameters, white matter signals, cerebrospinal fluid signals, and global mean signal were regressed out (Chao-Gan and Yu-Feng, 2010), and subjected to bandpass filtering (0.01–0.1 Hz).

After completing preprocessing, an analysis of the ANS modulation network was conducted. We selected distinct structures located in the brainstem, which are functionally associated with the regulation of the ANS, as the ROIs for FC analysis. The coordinates of the bilateral RVLM, CVLM, NTS, DMV and NA obtained from previous literature would be used as the center of the ROIs (Gerlach et al., 2019), and a radius 3 mm spherical area was defined as the ROIs. However, due to the proximity of the reference coordinates for NTS and NA, the analysis results of NTS were used as the primary reference. Similarly, the left and right localizations of DMV were also in close proximity, and thus, their analysis results were combined into one (Table 2).

**Table 2:** Reference coordinates of each region of interest (ROI).

ROIs		Coordinate		
		*X*	*Y*	*Z*
RVLM	ROI 1	7	−34	−45
	ROI 2	−7	−34	−45
CVLM	ROI 3	6	−38	−55
	ROI 4	−6	−38	−55
NTS/NA	ROI 5	5	−42	−48
	ROI 6	−5	−42	−48
DMV	ROI 7	1	−45	−54

ROI, region of interest; RVLM, rostral ventrolateral medulla; CVLM, caudal ventrolateral medulla; NTS, nucleus of the solitary tract; DMV, dorsal motor nucleus of the vagus; NA, nucleus ambiguus.

The time-series activity in the ROI was averaged and correlated with other brain regions by voxel-by-voxel manner. The resulting r value was standardized by converting it to a z value using Fisher’s r-to-z transformation to measure the FC strength between the ROI and other brain regions. This approach would help understand any changes in the brain’s autonomic neural regulation network.

### 2.7. Statistical analysis

For statistical analysis, we used SPSS version 22 to examine the demographic data variability (e.g., age, gender, BMI) between groups using ANOVA or Chi-square test to assess group comparability. In regards to brain imaging, we utilized SPM12 software for the analysis. The study employed a flexible factorial design with between-subject factors for groups and subject, and a within-subject factor for time. Our primary focus was on the effect of the intervention over time, so the statistical model included the interaction effect between the group and time factors, as well as the main effect of all three factors. To identify changes related to the intervention, a difference of differences analysis was performed between groups and times (i.e., experimental [post-pre]-control [post-pre]) using t-contrasts in the model. In addition, correlation analyses were performed to assess the relationships between the FC maps of ROIs and both the percentage of high-frequency (HF; 0.15-0.4Hz) power of HRV as well as the ratio of low-frequency (LF; 0.04-0.15 Hz) power percentage to HF power percentage of HRV, respectively. Given the frequently observed limited statistical power in fMRI studies (Cremers et al., 2017), the changes were deemed as significant if the family-wise error rate corrected cluster *p* < 0.05 (the cluster forming threshold is in uncorrected voxel *p* < 0.005). A significance level of *p* < 0.05 would be used to determine statistical significance in other analyses.

## 3. Results

### 3.1. Subjects

We recruited 51 healthy subjects in the final and separated into 3 groups in randomize sequence. Among them, there were 31 female subjects and 20 male subjects. The subjects aged 20 to 29 accounted for the most, 26 subjects in total, accounting for 51%; the BMI distribution of subjects was relatively average, 20.0 to 21.4 kg/cm^2^ accounted for the most, a total of 19 people, accounting for 37%. The remaining basic information of the subjects, including gender, age, and BMI distribution, is shown in Table 3 for details. According to the findings presented in Table 3, no significant differences were observed between the groups.

**Table 3:** Demographic data of the study population.

	Set 1	Set 2	Set 3	*p*-value
	0 J/cm^2^	7.96 J/cm^2^	23.87 J/cm^2^	
	*N* = 16	*N* = 18	*N* = 17	
Sex				0.674
Female	11	11	9	
Male	5	7	8	
*Age, years*				
20–29	10	7	9	
30–39	3	9	7	
40–49	2	2	1	
> 50	1	0	0	
Mean ± SD	29.25 ± 9.01	31.39 ± 6.71	30.94 ± 6.57	0.686
*BMI (kg/M^2^)*				
18.5–19.9	3	6	2	
20.0–21.4	7	8	4	
21.5–23.0	6	1	6	
23.0–24.0	0	3	5	
Mean ± SD	20.91 ± 1.38	20.89 ± 1.56	21.85 ± 1.45	0.103

BMI, Body Mass Index; SD, standard deviation.

### 3.2. Changes in FC maps of each ROIs

**Figure 2:**
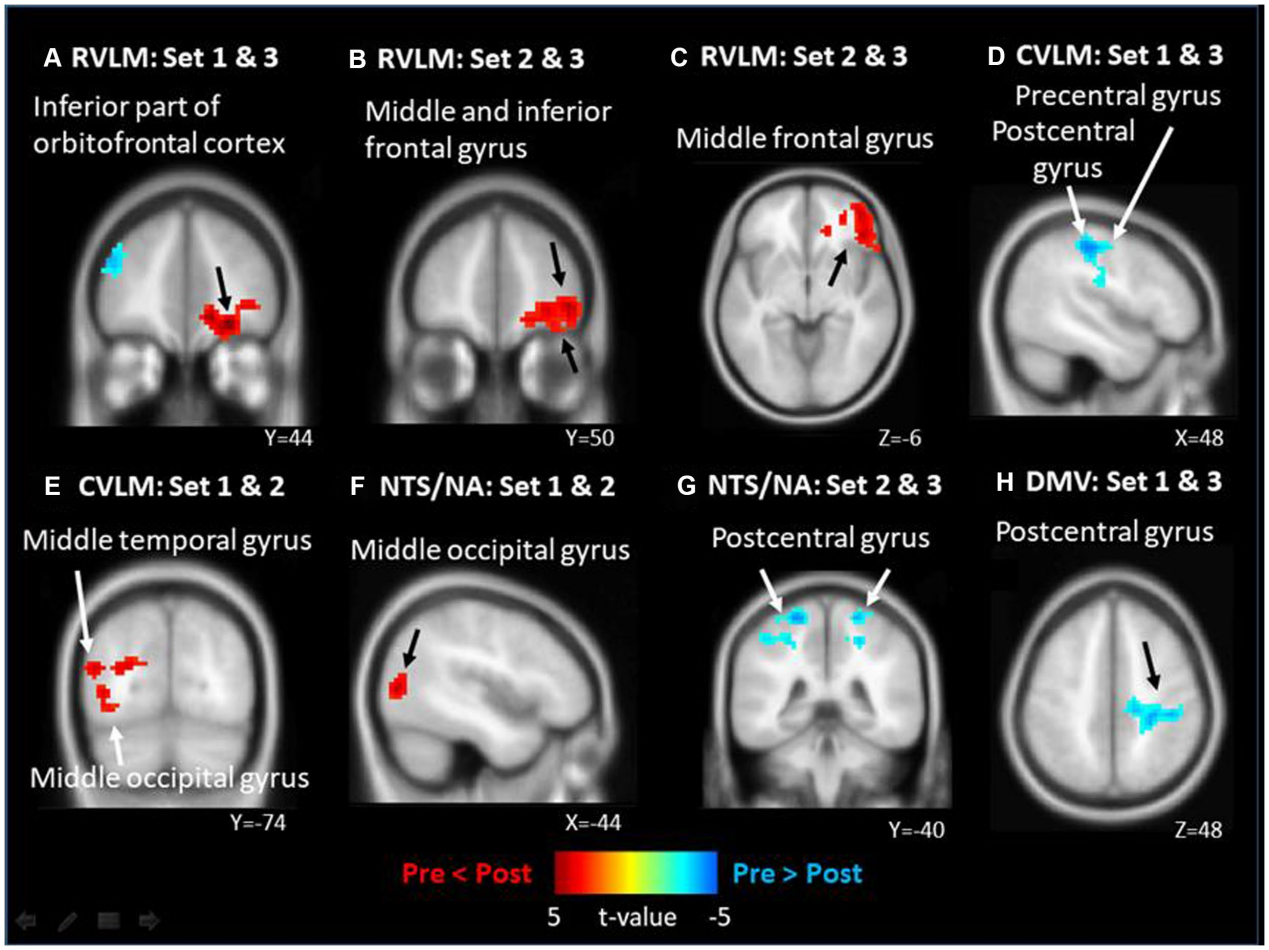
The significant functional connectivity (FC) changes of different brainstem nucleus after different energy density of laser acupuncture. **(A)** right side RVLM: FC enhanced in inferior part of orbitalfrontal cortex; **(B)** right side RVLM: FC enhanced in middle and inferior frontal gyrus; **(C)** left side RVLM: FC enhanced in middle gyrus; **(D)** right side CVLM: FC weakened in pre/post central gyrus; **(E)** left side CVLM: FC enhanced in middle temporal and occipital gyrus; **(F)** left side NTS/NA: FC enhanced in middle occipital gyrus; **(G)** left side NTS/NA as: FC weakened in bilateral postcentral gyrus; **(H)** DMV: FC weakened in postcentral gyrus of right side.

FC Maps of fMRI analysis results are shown in Figure 2. On the nucleus implicated in the regulation of the SNS, it is evident that the right RVLM leads to more significant FC enhancement in the inferior part of orbitofrontal cortex (OFC) following intervention in the 23.87 J/cm^2^ group when compared to the control group (Figure 2A). Additionally, the FC of right middle frontal gyrus (MFG) were more enhanced to bilateral RVLM after 23.87 J/cm^2^ intervention compared with group 7.96 J/cm^2^ (Figures 2B,C). Conversely, the FC of right CVLM is more weakened between the precentral and postcentral gyri in the 23.87 J/cm^2^ group compared to the 7.96 J/cm^2^ group (Figure 2D). The FC of left CVLM enhanced more is observed in the left middle temporal gyrus (MTG) and middle occipital gyrus (MOG) in the 7.96 J/cm^2^ group compared to the control group (Figure 2E).

Similarly, in other nuclei, such as those involved in the regulation of integration and the PNS, more enhanced FC is observed between left NTS/NA and MOG in the 7.96 J/cm^2^ group compared to the control group (Figure 2F). Otherwise, more weakened FCs is observed between both left NTS/NA and DMV and the postcentral gyrus after 23.87 J/cm^2^ interventions regardless of comparison to the 7.96 J/cm^2^ group of both sides (Figure 2G) or the control group (Figure 2H). Table 4 reports the details of observed significant changes in FCs in the brain for each ROI following stimulation with varying EDs.

**Table 4:** The results of significant differences in the functional connections (FCs) of each region of interests (ROIs) and brain.

Pre < Post										Pre > Post									
ROIs	Side	Anatomical area	BA	Size	*t*-max	Cluster *p*-value	Coordinate			ROIs	Side	Anatomical area	BA	Size	*t*-max	Cluster *p*-value	Coordinate		
						(FWE-corrected)	*x*	*y*	*z*							(FWE-corrected)	*x*	*y*	*z*
RVLM		Set 1 v.s. Set 3								RVLM									
	R	R Sup. Front. Gyrus	–	170	4.76	0.046	24	45	−15										
		Set 2 v.s. Set 3									None Significant								
	R	R Mid./Inf. Front. Gyrus	10/47	353	4.29	0.001	42	54	−6										
		Set 2 v.s. Set 3																	
	L	R Mid. Front. Gyrus	10/47	248	4	0.006	45	30	−3										
CVLM		Set 1 v.s. Set 2								CVLM		Set 1 v.s. Set 3							
	L	L Mid. Temp./Occi. Gyrus	39/19	207	3.96	0.017	−48	−75	18		R	R Post/Precentral Gyrus	40/4	420	5.09	< 0.001	42	−30	48
NTS/NA		Set 1 v.s. Set 2								NTS/NA		Set 2 v.s. Set 3							
	L	L Mid. Occi. Gyrus	39	178	4.36	0.035	−42	−78	6		L	R Postcentral Gyrus	2/5	184	3.92	0.03	24	−39	66
												L Postcentral Gyrus	2/3/4	257	4.63	0.005	−21	−36	66
DMV	None Significant									DMV	–	Set 1 v.s. Set 3							
												R Postcentral Gyrus	40/2	188	3.97	0.027	42	−30	48

ROI, region of interest; BA, Brodmann area; FEW, family wise error; RVLM, rostral ventrolateral medulla; CVLM, caudal ventrolateral medulla; NTS, nucleus of the solitary tract; DMV, dorsal motor nucleus of the vagus; NA, nucleus ambiguus; R, right; L, left; Sup., superior; Mid., middle; Inf., inferior; Frot., frontal; Temp., temporal; Occi., occipital.

### 3.3. Correlation between FC maps of nucleus and HRV

We further employed PPG-recorded HRV indices, specifically HF% representing PNS activity and LF/HF ratio reflecting SNS activity. These indices were subjected to correlation analysis with FC image in each group, aiming to uncover potential relationships between HRV indices and FC alterations.

**Figure 3:**
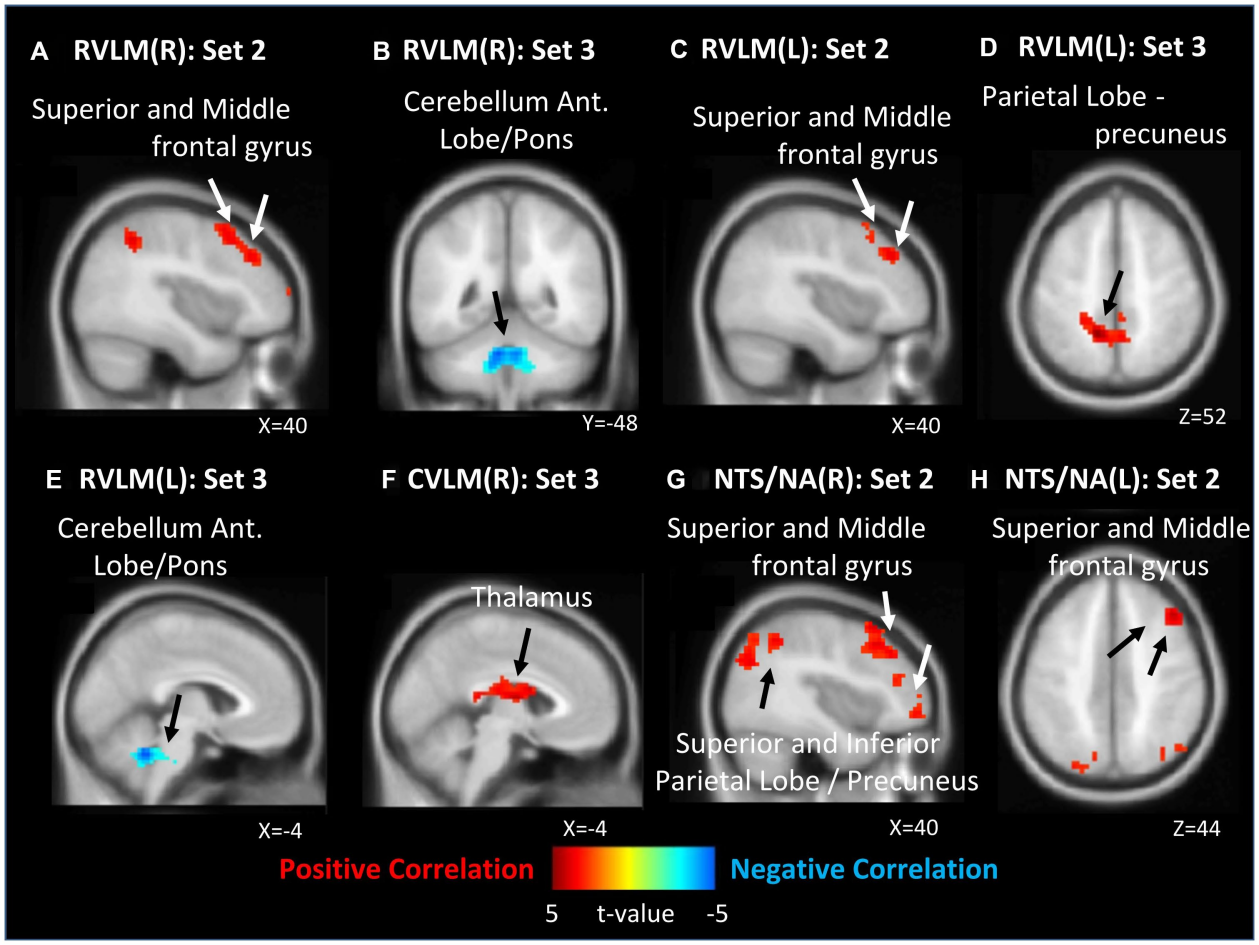
Correlation between functional connection (FC) maps of nucleus and percentage of high frequency (HF). **(A)** right side RVLM: positive correlated in superior and middle frontal gyrus; **(B)** right side RVLM: negative correlated in cerebellum anterior lobe near pons; **(C)** left side RVLM: positive correlated in superior and middle frontal gyrus; **(D)** left side RVLM: positive correlated in precuneus of parietal lobe; **(E)** left side RVLM: negative correlated in cerebellum anterior lobe near pons; **(F)** right side CVLM: positive correlated in thalamus; **(G)** right side NTS/NA: positive correlated in superior, middle frontal gyrus, superior, inferior precuneus of parietal lobe; **(H)** left side NTS/NA: positive correlated in superior and middle frontal gyrus.

In the analysis of all ROIs and HF%, 0 J/cm^2^ group exhibited no significant correlation with the HF% index. In the 7.96 J/cm^2^ group, significant positive correlations were observed in the superior and middle frontal gyrus between HF% and FCs of bilateral RVLM (Figures 3A,C) and NTS/NA (Figures 3G,H). Furthermore, positive correlations were also observed in superior, inferior parietal lobe, and precuneus in FCs of right NTS/NA (Figure 3G). Conversely, 23.87 J/cm^2^ group revealed significant negative correlations in the cerebellum adjacent to the pons between HF% and FCs of bilateral RVLM (Figures 3B,E). Additionally, a positive correlation emerged in the precuneus of the parietal lobe within the FC of the left RVLM (Figure 3D), as well as in the thalamus area in FC of the right CVLM (Figure 3F).

**Figure 4:**
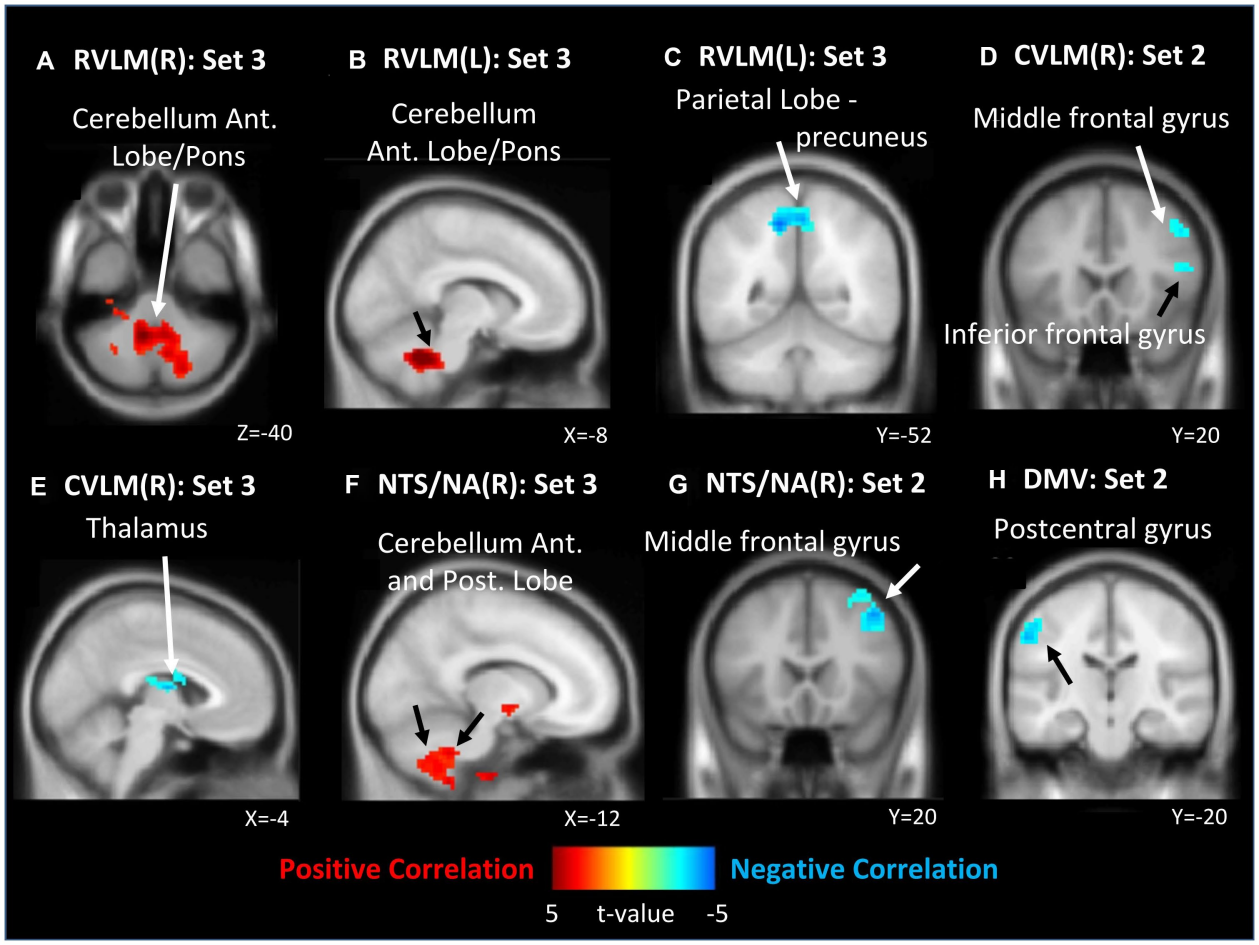
Correlation between functional connectivity (FC) maps of nucleus and low frequency (LF) to high frequency (HF) ratio. **(A)** right side RVLM: positive correlated in cerebellum anterior lobe near pons; **(B)** left side RVLM: positive correlated in cerebellum anterior lobe near pons; **(C)** left side RVLM: negative correlated in precuneus of parietal lobe; **(D)** right side CVLM: negative correlated in middle and inferior frontal gyrus; **(E)** left side RVLM: negative correlated in cerebellum anterior lobe near pons; **(F)** right side CVLM: negative correlated in thalamus; **(G)** right side NTS/NA: negative correlated in middle frontal gyrus; **(H)** DMV: negative correlated in postcentral gyrus.

In contrast, within the analysis of all ROIs and LF/HF ratio, 0 J/cm^2^ still exhibited no significant correlation. In 7.96 J/cm^2^ group, significant negative correlations were observed in the middle and inferior frontal gyrus between the LF/HF ratio and FCs of the right CVLM (Figure 4D) and NTS/NA (Figure 4G). Besides, a negative correlation in left postcentral gyrus has also been disclosed between the LF/HF ratio and FC of the DMV (Figure 4H). In 23.87 J/cm^2^ group, positive correlations were identified in the cerebellum adjacent to the pons between LF/HF ratio and FCs of bilateral RVLM (Figures 4A,B). Conversely, a negative correlation was noted in the precuneus of the parietal lobe to FC of left RVLM (Figure 4C), along with a negative correlation in the thalamus in relation to the FC of the right CVLM (Figure 4E). Furthermore, a positive correlation was observed in the cerebellum adjacent to the pons concerning the FC of right NTS/NA (Figure 4F). For the rest of the analysis results and details, please refer to the results in Table 5 (HF%) and Table 6 (LF/HF ratio).

**Table 5:** Correlation results between functional connection (FC) maps of different regions of interest (ROIs) and percentage of high frequency (HF).

HF									
ROIs	Side	Anatomical area	BA	Size	*t*-max	Cluster *p* value	Coordinate		
						(FWE-corrected)	*x*	*y*	*z*
*Positive correlation*									
RVLM		**Set 2**							
	R	R Sup./Mid. Front. Gyrus	9/46	231	4.05	0.01	42	18	48
		**Set 2**							
	L	R Sup./Mid. Front. Gyrus	9	165	3.5	0.046	39	36	36
		**Set 3**							
	L	L Pariet. Lobe - Precuneus	7	254	4.49	0.005	−12	−48	51
CVLM		**Set 3**							
	R	L Thalamus	–	435	4.08	< 0.001	−3	−6	12
NTS/NA		**Set 2**							
	R	R Sup./Inf. Pariet. Lobule Pariet. Lobe – Precuneus	7/19/39	219	3.97	0.013	36	−72	36
		R Sup./Mid. Front. Gyrus	8/9/10	310	3.94	0.002	36	21	57
		**Set 2**							
	L	R Sup./Mid. Front. Gyrus	8/9	183	4.22	0.03	42	24	48
*Negative correlation*									
RVLM		**Set 3**							
	R	Dentate nucleus/Pons	–	320	5.3	0.002	−6	−51	−33
		**Set 3**							
	L	Dentate nucleus/Pons	–	406	5.79	< 0.001	−6	−48	−33

HF, high frequency; ROI, region of interest; BA, Brodmann area; FEW, family wise error; RVLM, rostral ventrolateral medulla; CVLM, caudal ventrolateral medulla; NTS, nucleus of the solitary tract; NA, nucleus ambiguus; R, right; L, left; Sup., superior; Mid., middle; Frot., frontal; Pariet., parietal.

**Table 6:** Correlation results between functional connection (FC) maps of different regions of interest (ROIs) and low frequency (LF) to high frequency (HF) ratio.

LF/HF ratio									
ROIs	Side	Anatomical area	BA	Size	*t*-max	Cluster *p* value	Coordinate		
						(FWE-corrected)	*x*	*y*	*z*
*Positive correlation*									
RVLM		**Set 3**							
	R	Dentate nucleus/Pons	–	644	5.37	< 0.001	−6	−51	−33
		**Set 3**							
	L	Dentate nucleus/Pons	–	777	6.01	0.001	−9	−51	−33
NTS/NA		**Set 3**							
	R	Cerebellum Ant./Post. Lobe	–	380	4.27	< 0.001	−30	−60	−51
*Negative correlation*									
RVLM		**Set 3**							
	L	L Pariet. Lobe - Precuneus	7	302	4.77	0.002	−12	−51	51
CVLM		**Set 2**							
	R	R Mid./Inf. Front. Gyrus	−−/45	201	3.78	0.02	36	12	39
		**Set 3**							
	R	L Thalamus	–	206	3.85	0.018	−3	−9	12
NTS/NA		**Set 2**							
	R	R Mid. Front. Gyrus	9	233	4.26	0.009	42	21	45
DMV		**Set 2**							
	–	L Postcentral gyrus	3	163	3.89	0.047	−54	−18	39

LF, low frequency; HF, high frequency; ROI, region of interest; BA, Brodmann area; FEW, family wise error; RVLM, rostral ventrolateral medulla; CVLM, caudal ventrolateral medulla; NTS, nucleus of the solitary tract; DMV, dorsal motor nucleus of the vagus; NA, nucleus ambiguus; R, right; L, left; Ant., anterior; Post., posterior; Mid., middle; Inf., inferior; Frot., frontal; Pariet., parietal.

## 4. Discussion

The results of this study demonstrate that different EDs of LA at PC6 produce varying brain responses in ANS modulation network. Furthermore, the findings, as revealed by fMRI analysis, indicate that LA interventions with different EDs at PC6, ROIs known to modulate the ANS, produce statistically significant variations in FCs with different brain regions after intervention. The correlation analysis outcomes also reveal a statistically significant association between alterations in FC and variations in HRV indices. The results depicted in Figures 3, 4 reveal that both the 7.96 J/cm^2^ and 23.87 J/cm^2^ exhibit opposing correlation patterns in numerous brain regions, especially middle frontal gyrus in 7.96 J/cm^2^ group, cerebellum adjacent to the pons, and precuneus of the parietal lobe in 23.87 J/cm^2^ group.

As compared to the control group, the ROI analysis conducted using right RVLM revealed a significant increase in FC at the right inferior part of OFC following 23.87 J/cm^2^ ED stimulation (Figure 2A). These findings suggest that LA at this ED may modulate the neural pathways linking RVLM and OFC, potentially influencing autonomic regulation and associated processing. Previous research has suggested that the OFC plays a role in the processing of olfactory and gustatory information (Rolls, 2004), but recent research has expanded our understanding of the OFC’s function, highlighting its involvement not only in sensory processing but also in the regulation of visceral responses. The lateral portion of the OFC has been identified as the sensory network, responsible for processing olfactory, gustatory, and visual information, while the medial portion is considered the visceromotor network, which connects to the regulatory nucleus of the ANS and the PAG (Rudebeck and Murray, 2011). Recent research has established a correlation between ANS dysfunction, decreased HRV, and shrinkage of the OFC (Koenig et al., 2021). This decline in HRV intensifies progressively with age, surpassing the typical effects of aging (Koenig et al., 2021). Notably, intriguing findings have emerged regarding the potential of biofeedback training targeting HRV to induce an increase in cortical thickness, particularly in regions such as the OFC (Yoo et al., 2022). These new insights into OFC function have important implications for advancing our knowledge of the complex neural networks underlying autonomic regulation and visceral control.

Notably, some investigations have suggested that LA interventions with EDs within the 10–30 J/cm^2^ range may produce more significant analgesic effects (Cronshaw et al., 2019). The analgesic effects and underlying mechanisms of stimulating PC6 or the median nerve have been extensively studied and confirmed in numerous reports (Chen et al., 2018; Xiao et al., 2019; Lee et al., 2021). In conjunction with the findings of this study, we demonstrated that stimulation with an ED of 23.87 J/cm^2^ can increase the FC of OFC, and it may be posited that these observations provide a potential connection for the TCM to use of PC6, or median nerve, in the treatment of pathologies or clinical manifestations associated with discomfort in organ functionality such as palpitations, vomiting, and gastrointestinal problem (Fu et al., 2014). Furthermore, some investigations have suggested that the post-stimulation effects of PC6 may be closely associated with the modulation of sympathovagal balance (Jesus et al., 2021). This notion has been further supported by our correlation analysis. Following the 23.87 J/cm^2^ group intervention, several ROIs exhibit a negative correlation between the HF% index correlation to specific areas such as the pons and the anterior lobe of the cerebellum (Figures 3B,E). Additionally, there is a positive correlation of LF/HF ratio analysis to the same regions (Figures 4A,B,F).

Upon analyzing the right CVLM (Figure 2D), left NTS/NA (Figure 2G), and DMV (Figure 2H), it was observed that FCs within the postcentral gyrus of parietal lobe were attenuated in the 23.87 J/cm^2^ group, when compared to both the 7.96 J/cm^2^ group and the control group. The postcentral gyrus contains the primary somatosensory cortex. This region perceives various somatic sensations from the body, including touch, pressure, temperature, and pain (DiGuiseppi and Tadi, 2023). The observed reduction in FC in this region may indicate a corresponding decline in somatosensory ability of specific or non-specific body parts following LA stimulation at an ED of 23.87 J/cm^2^. This finding is consistent with previous studies examining the effects of PC6 stimulation and supports current research on the timing of clinical selection of PC6 (Fu et al., 2014; Chen et al., 2018; Xiao et al., 2019; Lee et al., 2021). Regrettably, no significant alterations in the postcentral gyrus were discerned in the analysis of HF% correlation across all groups. However, it is noteworthy that both 23.87 J/cm^2^ and 7.96 J/cm^2^ group exhibited a positive correlation to the parietal lobe and the precuneus (Figures 3D,G), which is in close proximity to the postcentral gyrus. In the analysis of LF/HF ratio correlation, a negative correlation was observed in postcentral gyrus in 7.96 J/cm^2^ group (Figure 4H). This result may offer partial support for the observed phenomena within each group of FC variations.

The right MFG has been suggested to act as a convergence point between the dorsal and ventral attentional networks. Specifically, it has been hypothesized that the MFG functions as a circuit-breaker, interrupting the ongoing endogenous attentional processes of the dorsal network and redirecting attention to an exogenous stimulus (Japee et al., 2015). Regardless of whether the left or right RVLM is selected for analysis (Figures 2B,C), the observed enhancement of FC in the MFG of the 23.87 J/cm^2^ group, relative to the 7.96 J/cm^2^ group, may provide some support for individual-specific or non-specific body parts changes in attentional shifting. Combining with the correlation analysis result, 7.96 J/cm^2^ group, not 23.87 J/cm^2^ group, exhibit a positive correlation in several ROIs between the HF% index and MFG (Figures 3A,C,G,H). Additionally, there is a negative correlation between the LF/HF ratio indicator and MFG (Figures 4D,G). The ED 7.96 J/cm^2^ may indeed influence the responses of the MFG, and further elucidation of the regulatory relationship in this context may be warranted.

Furthermore, several scholars have noted that the MTG play important roles in supporting various cognitive processes, including language and semantic memory processing, as well as visual perception and multimodal sensory integration (Onitsuka et al., 2004). Upon analyzing the left CVLM, it was observed that the 7.96 J/cm^2^ group exhibited greater FC with the left MTG than the control group. A few literatures have indicated that the MOG is involved in auditory and spatial processing (Renier et al., 2010). In our study, analysis of the left CVLM and left NTS/NA revealed that the 7.98 J/cm^2^ group exhibited increased FC with the MOG relative to the control group. Nevertheless, further exploration is required to determine whether changes in MOG are indicative of or correlated with changes in the FC of other brain regions.

In addition to the discourse on the functions of the respective cerebral regions, several related scholarly publications have presented noteworthy viewpoints. Presently, existing literature demonstrates that the stimulation of the median nerve exhibits the capacity to alleviate symptoms of nausea and vomiting (Arnberger et al., 2007; Kim Y. H. et al., 2011). This promoting effect is understood to be mediated through the vagus nerve pathway, particularly involving the integration of DMV and NTS (Maharjan et al., 2019). Furthermore, scholars such as Maharjan et al. have proposed that stimulating the median nerve also influences the activity of the OFC (Maharjan et al., 2019), and it has been empirically validated that the manipulation of the transcutaneous auricular vagus nerve stimulation could elicit discernible alterations in the FC of the OFC as detected by fMRI (Sun et al., 2022). These published evidences posit that the vagus neural network serves as a potential intermediary for transmitting the impact of median nerve stimulation on the OFC (Maharjan et al., 2019).

In further exploring the relationship between the sensorimotor cortex and the neural nuclei related to ANS regulation. In the early investigations, direct anterograde projections from the sensorimotor cortex (SMC) to the NTS, DMV, and RVLM have been experimentally verified with neuroanatomy basis (Sequeira et al., 2000). Traditionally, RVLM has been attributed to receive signals from the NTS via the CVLM when discussing the nucleus involved in ANS regulation (Albaghdadi, 2007). However, this study provides evidence that the SMC can independently modulate both the NTS and RVLM, suggesting a distinct regulatory influence of the SMC on these nuclei (Sequeira et al., 2000). In previous animal experiments, it has been discovered that electrical stimulation of the SMC of the brain has the potential to decrease mean arterial pressure by approximately 4-7 mmHg (Sequeira et al., 2000). These findings indicate that an appropriate level of SMC stimulation may effectively inhibit SNS activity. In our study, when the group receiving higher ED 23.87 J/cm^2^ stimulation was examined, a significant decrease in FC was observed between the precentral gyrus and postcentral gyrus (Figures 2D,G,H), in comparison to the other groups. This observation suggests that the dosage of stimulation administered might result in an enhanced of SNS activity performance. Previous experiment already revealed that the connection between the RVLM and corticospinal nerve bundles is more robust compared to the connection between the NTS and DMV (Sequeira et al., 2000). This suggests that the descending neural pathway originating from the SMC to the RVLM and corticospinal has its own foundation and distinctiveness and plays a more prominent role in ANS regulation, especially the sympathetic part, which may also be linked to the unique findings observed in the analysis results when the RVLM is used in our study.

Significant alterations in the FC between the OFC and the pre/postcentral gyrus have been documented in individuals with chronic ankle instability (CAI) (Shen et al., 2022). In the CAI population, a comparative analysis against the healthy control group reveals increased FC between the pre/postcentral gyrus, the sensorimotor network; while a concomitant decrease in FC is observed between the OFC and ACC, which associated with emotion or pain processing (Shen et al., 2022). Although, these observed changes diverge from the observed in our study comprising healthy subjects, these findings indirectly confirmed that OFC exhibits a synergistic relationship with brain regions responsible for somatosensory regulation. Despite the abundance of credible empirical findings and perspectives, the underlying factors responsible for the FC between the OFC and the SMC, or even other region mentioned, remain to be elucidated. It is yet to be determined whether this connection is influenced by regulatory mechanisms involving structures such as the RVLM, DMV, or other unidentified structures. Alternatively, it may be attributed to direct signal transmission within the disparate cerebral cortex itself. Although these factors were not the primary focus of this study, we look forward to further research in order to facilitate a comprehensive understanding of the underlying mechanisms driving this connectivity.

Based on the design and results of this experiment, while there may be some shortcomings, it is imperative to recognize its significant impact as it makes three notable contributions. First, to our knowledge, this is the first study to examine the effects of different LA EDs in conjunction with fMRI, providing insights into the potential differential responses of the brain to various intensities. These results have significant implications for understanding the potential neural mechanisms underlying the therapeutic effects of LA, and may inform future clinical applications of this technique. Second, currently empirical medical research on LA predominantly emphasizes the clinical application and intervention effects of specific diseases, as well as comparing it with other intervention methods, such as traditional acupuncture. Our study diverges from this trend by recruiting individuals from the general population and investigating the potential physiological follow-up effects resulting from stimulation of different EDs, an area that has received comparatively little attention. Our achievement is not only consistent with the usage methodology and parameter settings recommended by prior related journals (Huang et al., 2009; Zein et al., 2018; Cronshaw et al., 2019; Chang et al., 2022), but also carries implications for the safety of LA application and the varying biological effects resulting from distinct dosages. Our findings provided a valuable foundation and reference for operators and researchers in developing future treatment strategies or research designs. Third, this study incorporates multiple ROIs associated with ANS and HRV in medulla, in an effort to achieve a thorough comprehension of the distinct responses of diverse brain regions to varying EDs of LA. This approach is implemented to avoid potential confounding errors and inaccuracies stemming from a single structure-based analysis, and to facilitate a more complete understanding of the observed effects.

Nevertheless, it is important to acknowledge certain limitations that should be taken into consideration. When conducting an analysis of research pertaining to RVLM, alterations in blood pressure (BP) are commonly employed as indicators of changes in the ANS or SNS (Duan et al., 2022; Toledo et al., 2022). Nevertheless, the consideration of BP changes was omitted in this study. This omission is primarily due to this study’s reliance on established empirical medical research findings, and it is deemed unnecessary to reiterate the impact of RVLM on the SNS and BP. Our team’s preliminary research already exhibits that distinct EDs can produce varying degrees of impact on the ANS (Chang et al., 2022), exhibiting a biphasic dose–response phenomenon. The primary aim of this study is to employ this premise as a means to investigate alterations in the FC between relevant brain regions in response to varying EDs of LA. That is, BP and other ANS indicators are not our concern. Another limitation of our study is the inability to investigate or interpret the underlying molecular mechanisms associated with the subsequent effects of LA. Currently, the clinical efficacy of LA is grounded in findings from relevant journals reporting on animal experiments. These studies suggest that the local impact of LA is linked to the generation and synthesis of mitochondrial ATP (Xu et al., 2017), as well as the regulation of norepinephrine, epinephrine (Falaki et al., 2014) and acetylcholine (Mafra de Lima et al., 2009). However, the research methods employed in this study are confined to examining changes in neural activity and do not allow for the exploration of molecular mechanisms. Nevertheless, the findings from our study may serve as supportive evidence or contribute to the foundational knowledge underpinning ongoing or future research in the realm of molecular aspects. Furthermore, varying LA parameter settings can lead to diverse intervention outcome, such as wavelength, probe size, output power, exposure time, output mode, and emission oscillation/resonance frequency. In our investigation, we choose to use dose, ED, as a criterion for discussion and analysis. While controlling other parameters, we varied the exposure time to manipulate the ED. However, it should be noted that adjustments to the output power and exposure time, as well as the selection of different probes, can also produce the same ED. It is also still uncertain whether alternative settings would yield similar or distinct outcomes, and research into these relevant parameters is presently limited. PC6 is not the only acupoint that has been proven to affect HRV or ANS. Acupoints, such as Danzhong (CV17), Shenmen (HT7), Hegu (LI4), and Sanyinjiao (SP6), can effectively modulate the ANS and have varying degrees of impact on the indicators of HRV (Kurono et al., 2011; Kim E. et al., 2011; Huang et al., 2015). Despite limited research on the effects of LA stimulation on the aforementioned acupoints, it remains unclear whether different doses of LA can induce distinct biological effects similar to those observed in PC6. Finally, various studies have demonstrated differences in neural activity of brain between healthy and unhealthy individuals (Esposito et al., 2018; Piao et al., 2021; Shen et al., 2022). The design and results of this study, encompassing factors like subject conditions, may be influenced when extrapolated to other healthy groups, such as athletes, postmenopausal women, or specific disease cohorts. The impact is unclear whether the observed changes of imagines would lead to similar or entirely divergent results.

## 5. Conclusion

Different dosages of LA have demonstrated varied regions of enhanced or weakened FC between nuclei related to the regulation of the ANS and other brain areas. Over all, the findings suggest that the higher ED intervention, 23.87 J/cm^2^, at PC6 resulted in significantly enhanced FC of the OFC, which is involved in regulating visceral sensation, compared to the control group. Moreover, compared to a lower ED intervention, 7.96 J/cm^2^, and the control group, the higher ED intervention resulted in reduced FC in the postcentral gyrus, which is associated with somatosensory regulation. The correlation analysis results further indicate significant associations between variations in HRV indicators, particularly HF% and LF/HF ratio induced by different LA EDs stimulation, and alterations in brain FC between groups. Additionally, it is noteworthy that the performance of SNS and PNS performance exhibits opposing correlation changes in specific areas. These results probably indicated that differences in EDs can impact clinical benefits and subsequent outcomes by modulating distinct neural pathways in the brain. The results of this pilot study may be a basis for further discussion of the potential effects and alterations of various LA parameters or EDs on other brain regions. Future studies could investigate the optimal parameters for LA interventions and how they may vary depending on different conditions. Additionally, studies could explore the long-term effects of LA using fMRI and other physiological markers to determine it’s yet to be explored potential.

## Data availability statement

The original contributions presented in the study are included in the article/Supplementary material, further inquiries can be directed to the corresponding authors.

## Ethics statement

The studies involving humans were approved by the Research Ethics Committee of China Medical University and Hospital. The studies were conducted in accordance with the local legislation and institutional requirements. The participants provided their written informed consent to participate in this study.

## Author contributions

Y-CC: Conceptualization, Formal analysis, Investigation, Visualization, Writing – original draft. C-MC: Data curation, Software, Writing – review & editing. I-SL: Resources, Supervision, Writing – review & editing. Y-CL: Supervision, Validation, Writing – review & editing. C-HT: Conceptualization, Data curation, Formal analysis, Funding acquisition, Investigation, Methodology, Project administration, Resources, Validation, Writing – review & editing.
